# Molecular mechanisms of deer antler in promoting osteogenic differentiation of human mesenchymal stem cells via JUN modulation

**DOI:** 10.3389/fimmu.2025.1550249

**Published:** 2025-05-29

**Authors:** Chengcheng Yu, Yinan Wu, Hanxu Huang, Xiumao Li, Jingkai Wang, Chao Liu, Yuanqing Shen, Donghua Huang, Ruofu Tang, Zhan Wang, Lifeng Jiang, Fangcai Li

**Affiliations:** ^1^ Department of Orthopedics, Second Affiliated Hospital, Zhejiang University School of Medicine, Hangzhou, China; ^2^ Zhejiang Cancer Hospital, Institute of Medical Research, Chinese Academy of Sciences, Hangzhou, China; ^3^ Rehabilitation Department, Second Affiliated Hospital, Zhejiang University School of Medicine, Hangzhou, China

**Keywords:** deer antler, osteogenic differentiation, JUN, bioinformatics analysis, machine learning, immune microenvironment modulation

## Abstract

**Background:**

Traditional Chinese medicine and food deer antler has been extensively used in bone regeneration, but its molecular mechanisms remain poorly understood. Preliminary investigations suggest deer antler contains bioactive compounds that influence osteogenic differentiation and immune microenvironments.

**Purpose:**

To elucidate the molecular mechanisms of deer antler in promoting human mesenchymal stem cell (hMSC) osteogenic differentiation, focusing on JUN downregulation and immune microenvironment modulation using bioinformatics and molecular docking approaches.

**Methods:**

Chemical components and targets were identified using the BATMAN-TCM database. Differentially expressed genes (DEGs) related to osteogenic differentiation were analyzed using Gene Expression Omnibus datasets. Gene Ontology (GO), KEGG enrichment, LASSO regression, and SVM-RFE were applied to identify key genes. A Protein-Protein Interaction (PPI) network was constructed to determine core genes. JUN expression was validated using independent datasets and ROC analysis. Immune cell infiltration was analyzed using CIBERSORT, examining JUN’s correlation with immune cells. Molecular docking explored JUN’s interaction with two active deer antler compounds.

**Results:**

The study identified 62 bioactive compounds and 1051 potential targets. DEGs analysis revealed 282 genes associated with osteogenic differentiation. Cross-analysis identified 43 overlapping genes, enriched in “response to mechanical stimulus” and “rheumatoid arthritis” pathways. Machine learning approaches highlighted 7 critical genes, with JUN emerging as the core gene. JUN levels were significantly decreased during osteogenic differentiation, showing high diagnostic accuracy (AUCs: 0.977-1.000). Immune cell analysis revealed JUN correlations with neutrophils, monocytes, eosinophils, M2 macrophages, and resting CD4+ T cells. Molecular docking confirmed strong binding affinities of JUN with Retinol (-8.1 kcal/mol) and Progesterone (-6.0 kcal/mol).

**Conclusions:**

The study provides a comprehensive molecular framework demonstrating JUN as a key molecule in hMSC osteogenic differentiation. Deer antler’s bioactive compounds, particularly Retinol and Progesterone, potentially exert therapeutic effects through targeted JUN modulation, offering novel insights into natural compound-mediated bone regenerative mechanisms.

## 1 Introduction

The restoration and healing of bones are essential for preserving skeletal health, especially in the context of fractures, osteoporosis, and other degenerative bone disorders (1, 2). Human mesenchymal stem cells (hMSCs) are crucial for this process, as their transformation into bone-forming cells is key to new bone tissue development (2, 3). Although our knowledge of the cellular and molecular basis of bone regeneration has grown, there is still a need for more effective treatments, especially in complex scenarios where there is a need to boost bone-forming capabilities and regulate the immune system (4, 5).

Deer antler (Cornu Cervi Pantotrichum), a well-known traditional Chinese medicine and food, is widely utilized for its potential to improve bone health and facilitate repair (6, 7). This natural remedy is abundant in bioactive components such as growth factors, peptides, and minerals, which are thought to contribute to its healing properties by promoting the development of bone tissue and adjusting the immune response (6, 8, 9). However, the molecular mechanisms underlying these effects remain poorly understood, posing a challenge to the scientific validation and clinical application of deer antler (10).

Recent advances in bioinformatics and high-throughput data analysis have provided valuable tools for investigating the complex interactions between traditional Chinese medicine and molecular targets (11). In particular, network pharmacology and machine learning techniques offer new opportunities to identify bioactive compounds, potential targets, and regulatory pathways associated with therapeutic effects (12). These computational approaches have revolutionized our understanding of traditional medicine mechanisms (13).

This study aims to elucidate the molecular mechanisms by which deer antler promotes osteogenic differentiation in hMSC, focusing on its effects on the key regulatory gene and the immune microenvironment. Employing bioinformatics, machine learning algorithms, immunological profiling, and molecular docking techniques, this study investigates the mechanisms by which the bioactive constituents of deer antler engage with molecular signaling cascades to potentiate osteogenic processes. These findings are expected to provide a scientific foundation for the therapeutic use of deer antler in bone-related disorders and contribute to the modernization of traditional Chinese medicine practices.

## 2 Materials and methods

### 2.1 Identification of bioactive compounds and potential targets of deer antler

The bioactive compounds of deer antler and their potential molecular targets were identified using the BATMAN-TCM database (14), a comprehensive platform for exploring traditional Chinese medicine and its pharmacological mechanisms. Potential targets were systematically extracted based on compound-target interactions with confidence score cutoff≥0.86 (LR=112.67) and adjusted p-value ≤ 0.05.

### 2.2 Collection and preprocessing of gene expression datasets

Gene expression profiles associated with osteogenic differentiation and stem cell proliferation were retrieved from the Gene Expression Omnibus (GEO) database (15). Five datasets (GSE80614, GSE100752, GSE12267, GSE28205 and GSE9451) were curated based on rigorous criteria pertinent to osteogenic differentiation and stem cell research. Subsequent data preprocessing encompassed normalization and batch effect correction, facilitated by the R packages limma (version 3.62.1) (16) and edgeR (v4.4.0) (17). Principal Component Analysis (PCA) was performed to visualize sample clusters and evaluate potential batch effects. The Combat algorithm (18) from the sva package (v3.54.0) (19) was applied to remove batch effects. Differentially expressed genes (DEGs) were identified using the criteria of |log2FoldChange| > 1 and adjusted p-value < 0.05. Data visualization was accomplished through hierarchical clustering heatmaps and volcano plots generated using the Complex Heatmap package in R (20). Detailed descriptions of datasets including sample size, cell source, induction conditions, and culture environments are summarized in **Supplementary Table S1**. To address dataset heterogeneity, the Combat algorithm from the sva package was used to perform batch correction. The efficacy of this correction was validated using PCA visualization.

### 2.3 Functional enrichment analysis

Gene Ontology (GO) (21) and Kyoto Encyclopedia of Genes and Genomes (KEGG) (22) pathway enrichment analyses were conducted using the ClusterProfiler package (v4.14.3) (23) in R. The intersection between DEGs and deer antler target genes was identified through Venn diagram analysis using the VennDiagram package (24). The resulting overlapping genes underwent further functional enrichment analysis to elucidate their roles in biological processes and molecular pathways.

### 2.4 Machine learning for key gene identification

Two complementary machine learning approaches were employed to identify key regulatory genes. Least Absolute Shrinkage and Selection Operator (LASSO) regression was implemented using the glmnet package (v4.1-8) (25), while Support Vector Machine Recursive Feature Elimination (SVM-RFE) was performed using the e1071 package (v1.7-16) (26). Genes identified by both methods with consistent selection frequencies > 80% were retained for subsequent analyses.

### 2.5 Protein-protein interaction network analysis

A PPI network was constructed using both STRING database (v11.5) (27) and GeneMANIA database (28) with an interaction confidence score threshold of 0.4. Core regulatory genes were determined based on the centrality metrics from both networks.

### 2.6 Validation of gene expression and diagnostic performance

Independent validation was performed using GEO datasets GSE18043 and GSE28074. These validation datasets underwent identical preprocessing procedures as the primary analysis. Receiver Operating Characteristic (ROC) curves were generated using the pROC package (v1.18.5) (29), with Area Under the Curve (AUC) values calculated to assess diagnostic accuracy.

### 2.7 Immune infiltration analysis

Immune microenvironment analysis was conducted using the Cell-type Identification By Estimating Relative Subsets Of RNA Transcripts (CIBERSORT) algorithm (30) with the LM22 signature matrix. Results were visualized using ggplot2 (v3.5.1) (31) for heatmaps and violin plots. Correlations between core regulatory gene expression and immune cell populations were assessed using Pearson correlation analysis, with significance threshold set at p < 0.05. All immune cell estimations were performed on batch-corrected gene expression data. Low-quality samples were excluded using LM22 matrix quality thresholds to ensure robust immune deconvolution.

### 2.8 Molecular docking analysis

Molecular interactions between core regulatory gene and deer antler bioactive compounds were investigated through molecular docking simulations. Three-dimensional structures of bioactive compounds were obtained from PubChem (32), while the core regulatory gene protein crystal structure was retrieved from the Protein Data Bank (33). Docking simulations were performed using AutoDock Vina (v1.5.6) (34), and molecular interactions were visualized using PyMOL (v3.1.0) (34).

### 2.9 Statistical analysis

All statistical analyses were performed in R (version 4.4.2) (35). Multiple testing corrections were implemented using the Benjamini-Hochberg method to control the false discovery rate (FDR). Statistical significance was set at p < 0.05 unless otherwise specified.

## 3 Results

### 3.1 Identification of bioactive compounds and potential targets in deer antler

Analysis through the BATMAN-TCM database (14) identified 62 bioactive compounds and 1,051 potential molecular targets in deer antler.

### 3.2 Identification of differentially expressed genes

**Figure 1:**
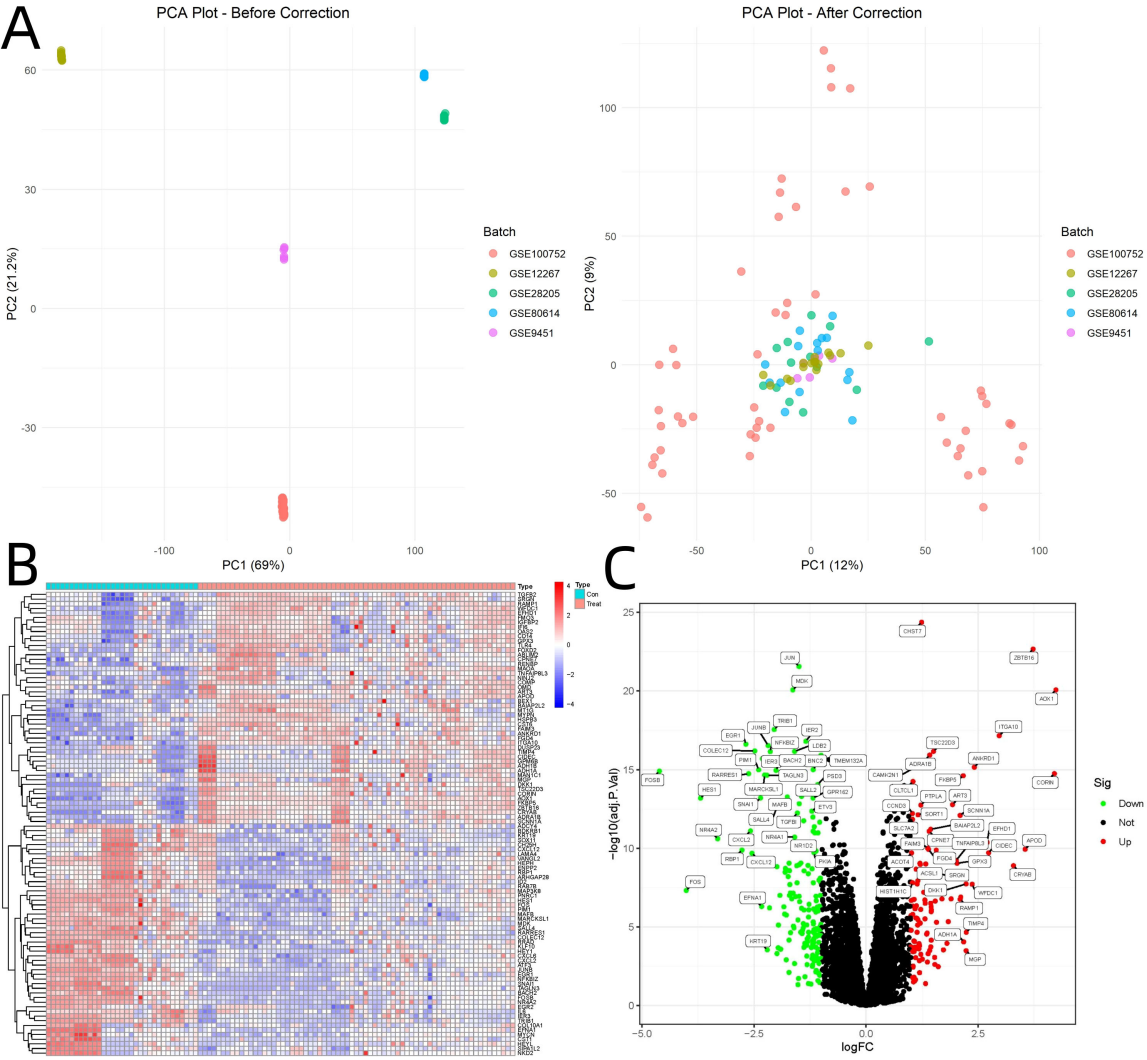
Visualization of Batch Correction and Differential Gene Expression Analysis. **(A)** PCA visualization before and after batch correction. **(B)** Hierarchical clustering analysis of GEO dataset. **(C)** Volcano plot.

PCA visualization before and after batch correction confirmed the effectiveness of the correction (**Figure 1A**). Analysis of GEO datasets yielded 282 DEGs associated with osteogenic differentiation (|log2FoldChange| > 1, adjusted p < 0.05). Hierarchical clustering analysis revealed distinct expression patterns between osteogenic differentiation and control groups (**Figure 1B**). Volcano plot visualization highlighted 112 upregulated and 170 downregulated genes (**Figure 1C**).

### 3.3 Overlapping genes and functional enrichment analysis

**Figure 2:**
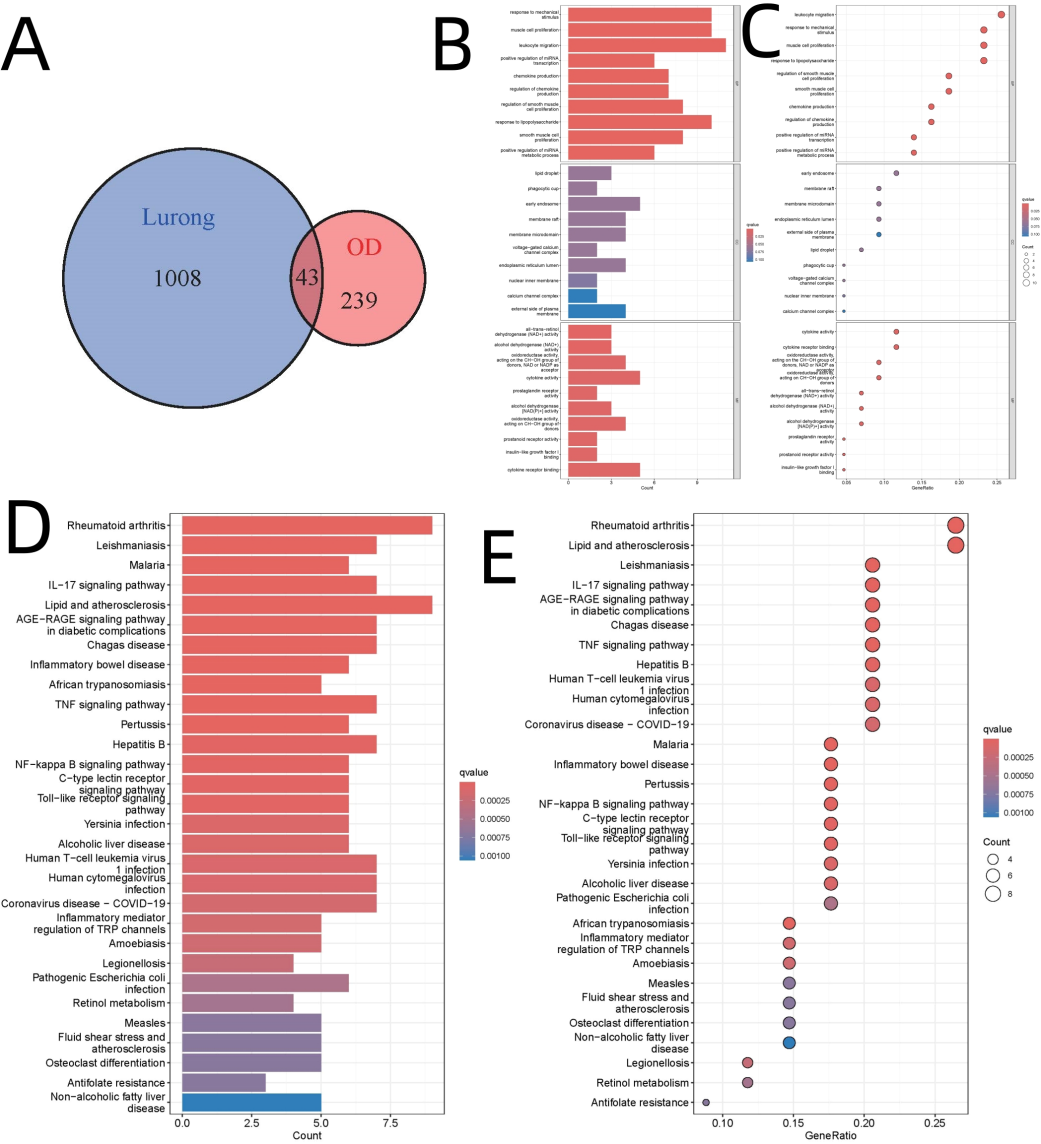
Integration of Deer Antler Targets with DEGs and Enrichment Analysis. **(A)** Venn diagram illustrating the integration of deer antler targets. **(B)** Bar diagram of GO analysis. **(C)** Bubble diagram of GO analysis. **(D)** Bar diagram of KEGG pathway analysis. **(E)** Bubble diagram of KEGG pathway analysis.

Upon integrating the deer antler-associated targets (n=1,051) with DEGs (n=282), we identified 43 intersecting genes, as depicted in **Figure 2A**. GO analysis revealed that the biological processes “response to mechanical stimulus” (GO:0009612, adjusted p-value=1.14E-07) and “muscle cell proliferation” (GO:0033002, adjusted p-value=1.99E-07) were significantly enriched, as illustrated in **Figures 2B, C**. KEGG pathway analysis indicated a pronounced enrichment in immune-modulatory pathways, particularly “rheumatoid arthritis” (hsa05323) and “lipid and atherosclerosis” (hsa0541), with adjusted p-values less than 3E-6, as shown in **Figures 2D, E**. These findings align with recent studies highlighting the mechano-immunological regulation of bone regeneration (36). Specifically, the enrichment of ‘rheumatoid arthritis’ pathway highlights the immunological basis of osteogenic modulation, given the role of T cells and monocytes in bone microenvironment remodeling and osteoclast activity.

### 3.4 Machine learning and PPI identifies JUN as a core gene

**Figure 3:**
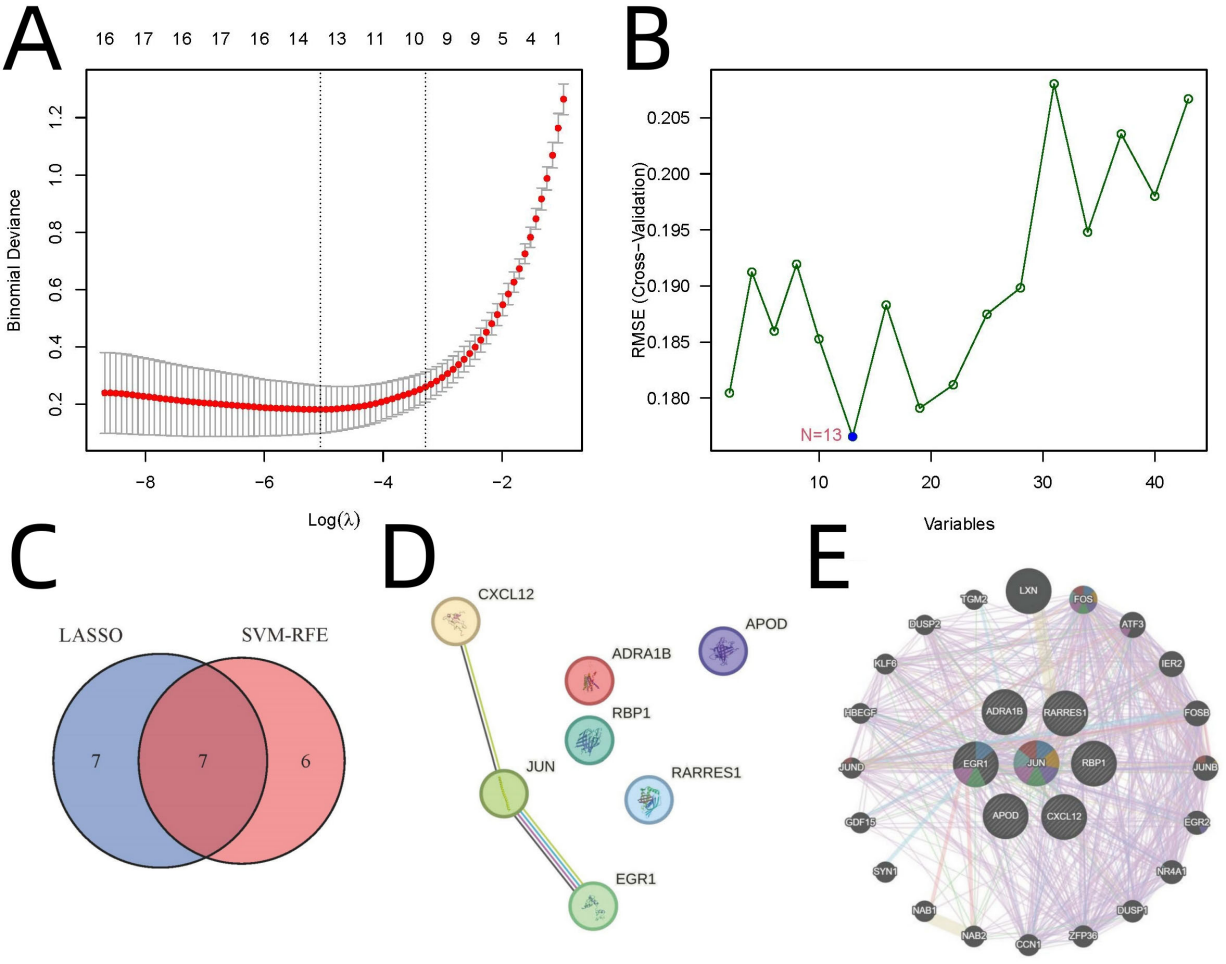
Identification of Key Regulatory Genes Using Machine Learning and PPI Analysis. **(A)** LASSO regression. **(B)** SVM-RFE analysis. **(C)** Venn diagram highlighting seven genes consistently identified by both methods. **(D)** PPI network analysis by STRING. **(E)** PPI network analysis by GeneMANIA.

Advanced machine learning approaches identified key regulatory genes from the 43 overlapping candidates. LASSO regression identified 14 genes (λ=1) (**Figure 3A**), while SVM-RFE yielded 13 genes (10-fold cross-validation accuracy=0.824) (**Figure 3B**). Seven genes were consistently identified by both methods: JUN, EGR1, ADRA1B, RARRES1, APOD, RBP1, and CXCL12 (**Figure 3C**). Protein-protein interaction (PPI) network analysis revealed JUN as the hub gene (**Figures 3D, E**). The LASSO regression was tuned using 10-fold cross-validation to select λ=1. SVM-RFE was configured with a linear kernel and optimized with 10-fold CV to achieve 82.4% accuracy. These settings are provided in the supplementary code.

### 3.5 Validation of JUN expression

**Figure 4:**
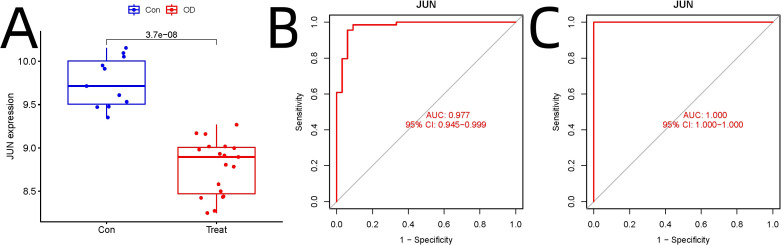
Independent Validation of JUN as a Key Regulator in Osteogenic Differentiation. **(A)** Independent validation. **(B)** ROC analysis in the experimental dataset. **(C)** ROC analysis in the validation dataset analysis.

Independent validation using GEO datasets GSE18043 and GSE28074 confirmed significant downregulation of JUN in osteogenic differentiation compared to hMSC controls (adjusted p=3.7E-08) (**Figure 4A**). ROC analysis demonstrated JUN’s robust diagnostic potential, with AUC values of 0.977 (95% CI: 0.945–0.999) in the experimental dataset (**Figure 4B**) and 1.000 (95% CI: 1.000–1.000) in the validation dataset (**Figure 4C**).

### 3.6 Immune infiltration analysis and JUN-immune cell correlations

**Figure 5:**
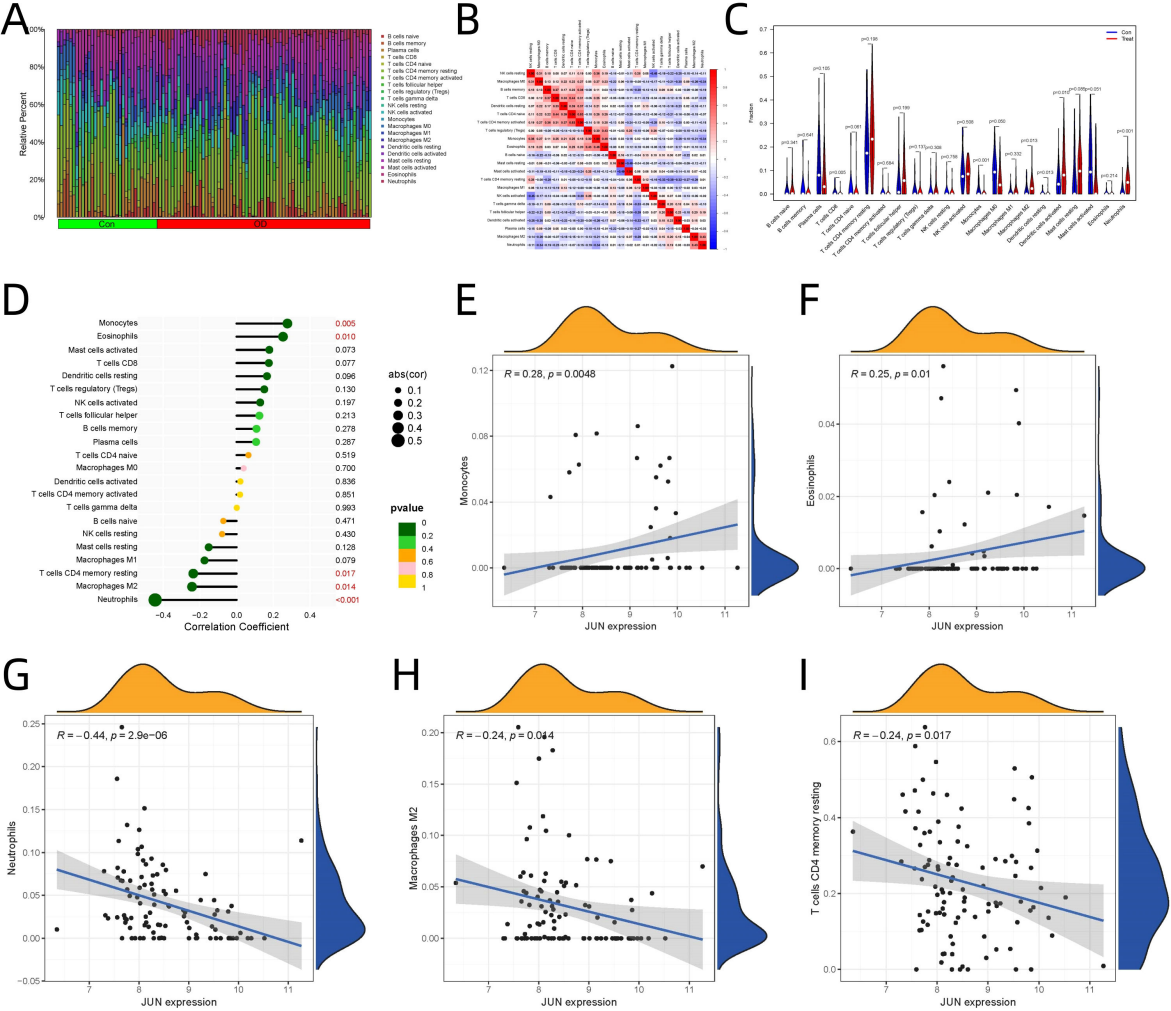
Immune Infiltration Analysis and Correlation of JUN Expression with Immune Cells. **(A)** Immune cell proportions between osteogenic differentiation and control groups. **(B)** Relationships among immune cell populations. **(C)** Specific immune populations between osteogenic differentiation and control groups. **(D)** JUN expression correlations with immune cell. **(E)** JUN’s role in monocyte. **(F)** JUN’s role in eosinophils. **(G)** JUN’s role in neutrophils. M2 Macrophages **(H)** JUN’s role in M2 macrophages. **(I)** JUN’s role resting memory CD4+ T cells.

CIBERSORT analysis revealed significant alterations in immune cell proportions between osteogenic differentiation and control groups (**Figure 5A**). The relationships between different immune cell populations based on their infiltration levels are illustrated (**Figure 5B**). Significant differences in immune cell populations were observed, as presented in **Figure 5C**: CD8+ T cells (p = 0.005), monocytes (p < 0.001), M2 macrophages (p = 0.013), neutrophils (p < 0.001), dendritic cells in both resting (p = 0.013) and activated states (p = 0.010).

JUN expression showed significant correlations with specific immune cell populations (**Figure 5D**):

Significant Positive Correlations

1. Monocytes (**Figure 5E**) exhibited the strongest positive correlation (R=0.277, p=0.005), suggests potential crosstalk between JUN signaling and monocyte recruitment/activation during osteogenic differentiation.
2. Eosinophils (**Figure 5F**) showed notable positive correlation (R=0.253, p=0.010), indicates possible involvement of eosinophil-mediated processes in the differentiation context.

Significant Negative Correlations

1. Neutrophils (**Figure 5G**) demonstrated the strongest negative correlation (R=-0.444, p<0.001), inverse relationship suggests potential suppressive effects during osteogenic differentiation.
2. M2 Macrophages (**Figure 5H**) showed significant negative correlation (R=-0.243, p=0.014), indicates a shift in macrophage polarization during differentiation.
3. Resting memory CD4+ T cells (**Figure 5I**) displayed negative correlation (R=-0.236, p=0.017), suggests potential immunomodulatory effects on T cell populations.

### 3.7 Molecular docking confirms JUN-bioactive compound interactions

**Figure 6:**
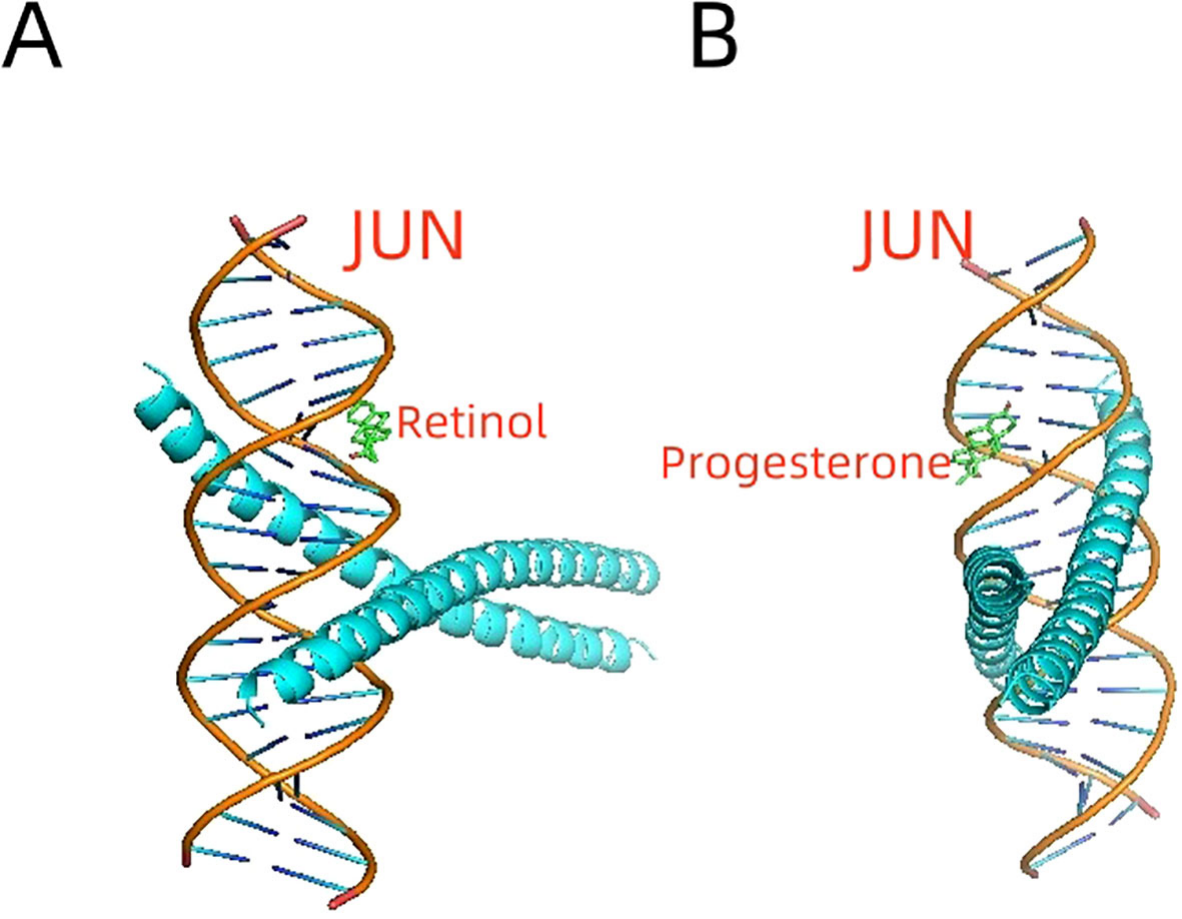
Molecular Docking Simulations of JUN Protein with Deer Antler-Derived Bioactive Compounds. **(A)** Retinol demonstrates a strong interaction with the JUN protein. **(B)** Progesterone shows a stable binding interaction with JUN.

Molecular docking simulations demonstrated strong interactions between the JUN protein and bioactive compounds derived from deer antlers. Specifically, retinol exhibited a binding energy of -8.1 kcal/mol (**Figure 6A**), while progesterone showed a binding energy of -6.0 kcal/mol (**Figure 6B**). These binding energy values indicate stable molecular interactions, suggesting that deer antler compounds may directly modulate JUN activity.

### 3.8 Summary of key findings

1. Identification of 62 bioactive compounds and 1,051 potential targets in deer antler.
2. Detection of 282 DEGs in osteogenic differentiation.
3. Discovery of 43 overlapping genes between deer antler targets and DEGs.
4. Identification of JUN as a core regulatory gene through machine learning and PPI.
5. Validation of JUN’s role through expression analysis and diagnostic performance.
6. Establishment of JUN’s relationship with immune cell populations.
7. Confirmation of molecular interactions between JUN and deer antler compounds.

## 4 Discussion

Bone regeneration represents a complex biological process orchestrated by intricate cellular signaling networks, immune system interactions, and environmental factors (37). This study presents a comprehensive investigation of deer antler’s molecular mechanisms in promoting human mesenchymal stem cell (hMSC) osteogenic differentiation. Our findings identify JUN as a pivotal regulatory molecule and demonstrate how deer antler’s bioactive compounds may facilitate bone regeneration through JUN-mediated pathways.

### 4.1 JUN as a central regulator of osteogenic differentiation

JUN, a key component of the activator protein-1 (AP-1) transcription factor family, orchestrates various cellular processes including proliferation, differentiation, and stress responses (38). Our analysis revealed significant downregulation of JUN during osteogenic differentiation, consistent with previous studies suggesting its role as a negative regulator of osteogenesis (39). The remarkable diagnostic performance of JUN (experimental AUC: 0.977; validation AUC: 1.000) establishes it as a robust biomarker for osteogenic differentiation. This downregulation appears to be a critical checkpoint in the osteogenic pathway, potentially facilitating the activation of pro-osteogenic transcriptional programs (40). However, the role of JUN was identified via bioinformatics analysis only. Functional validation such as gene knockdown or overexpression experiments are planned in follow-up studies to verify its causal role in osteogenesis.

### 4.2 Immune modulation and JUN’s role in the osteogenic microenvironment

The significant changes in immune cell composition suggest an active immune response during osteogenic differentiation. The increased proportion of CD8+ T cells, monocytes, and M2 macrophages aligns with previous findings showing their involvement in bone regeneration and remodeling (41). The presence of both resting and activated dendritic cells suggests ongoing immune surveillance and potential antigen presentation during the differentiation process (42).

The correlation between CD4+ T cell subsets indicate coordinated T cell responses, while the M2 macrophage-neutrophil correlation suggests a potentially orchestrated innate immune response (**Figure 5B**). These findings provide new insights into the immune microenvironment during osteogenic differentiation and may have implications for bone tissue engineering and regenerative medicine (43).

The transcription factor JUN plays crucial roles in immune response regulation through its differential association with various immune cell populations. Our correlation analysis reveals several significant relationships that align with and extend previous findings in the literature.

The positive correlation between JUN expression and monocytes supports previous studies demonstrating JUN’s essential role in monocyte differentiation and function (38, 44). A striking negative correlation was observed between JUN and neutrophils, representing the strongest relationship in our study. This finding suggests JUN may act as a negative regulator of neutrophil activation states. This observation is consistent with work by Behre et al. (45). The strong negative correlation might indicate a regulatory mechanism where JUN helps maintain neutrophil homeostasis by preventing excessive activation. The negative correlation with M2 macrophages provides interesting insights into JUN’s potential role in macrophage polarization. This relationship suggests that JUN might influence the balance between M1 and M2 phenotypes (46). The observed correlations with resting memory CD4+ T cells and eosinophils suggest broader immunomodulatory roles for JUN. The negative correlation with memory CD4+ T cells might reflect JUN’s involvement in T cell quiescence, as supported by Riera-Sans et al. (47).

Collectively, our results imply that the JUN protein serves as a central regulatory hub within the immune system, modulating the equilibrium among various immune cell subsets. The contrasting correlations observed with distinct myeloid cell types—positively associated with monocytes and negatively with neutrophils and M2 macrophages—suggest a potential role for JUN as a molecular determinant in the lineage commitment and functional specification of myeloid cells.

### 4.3 Bioactive compounds in deer antler target JUN

Utilizing the BATMAN-TCM database, our investigation revealed 62 bioactive compounds and 1,051 potential targets in deer antler. Molecular docking identified Retinol and progesterone as key interactors with the JUN transcription factor, with binding energies of -8.1 kcal/mol and -6.0 kcal/mol, respectively. Given Retinol’s established role in osteogenic differentiation and its stable interaction with JUN, it emerges as a promising candidate for bone health therapies (48). Progesterone boosts bone health via multiple mechanisms like regulating bone resorption and formation (49). Its significant binding affinity with JUN implies its role in bone regeneration. These molecular interactions establish a fundamental groundwork for the traditional function of deer antler in bone regeneration and its therapeutic uses.

### 4.4 Functional enrichment and pathway analysis

Functional enrichment analysis has identified a number of crucial biological processes and pathways that are significantly correlated with the bioactive components of deer antler. The prominent discoveries are as follows:

1. Response to Mechanical Stimulus (GO:0009612, p < 0.001): This indicates a function in mechanotransduction, which is an essential process in bone remodeling (50);
2. Rheumatoid Arthritis Pathway (hsa05323, p < 0.001): It underlines the potential immunomodulatory impacts that might sustain bone health under inflammatory circumstances (51);
3. Immune Modulation Pathways: This points to a cooperative interaction between immune responses and bone regeneration procedures (52).

These results align with current understandings of bone physiology, emphasizing mechanical and immunological pathways as the principal mediators of the therapeutic outcomes of deer antler.

### 4.5 Implications for bone regeneration therapies

The identification of JUN as a central regulator presents several therapeutic opportunities:

1. Development of targeted interventions focusing on JUN modulation (53);
2. Optimization of deer antler-derived compounds for therapeutic applications (6);
3. Integration with current stem cell-based therapies.

### 4.6 Study limitations and future directions

The study is subject to several limitations. Firstly, it depends on bioinformatics findings which call for experimental verification. Secondly, it only concentrates on particular compounds within the intricate composition of deer antlers. Thirdly, bulk RNA sequencing has limitations in analyzing immune cell heterogeneity. Future endeavors should prioritize *in vitro* and *in vivo* validations, investigate additional bioactive compounds, and employ single-cell RNA sequencing to achieve a more nuanced understanding of the immune microenvironment.

## 5 Conclusion

This study establishes JUN as a critical regulator in hMSC osteogenic differentiation and elucidates potential mechanisms through which deer antler bioactive compounds may promote bone regeneration. The findings provide a scientific foundation for the development of novel therapeutic strategies in bone regeneration.

## Data Availability

The original contributions presented in the study are included in the article/**Supplementary Material**. Further inquiries can be directed to the corresponding author.

## References

[1] Berni M Brancato AM Torriani C Bina V Annunziata S Cornella E. The role of low-level laser therapy in bone healing: systematic review. Int J Mol Sci. (2023) 24:(8). doi: 10.3390/ijms24087094 PMC10139216 37108257

[2] Yu XH Tang XY Gohil SV Laurencin CT. Biomaterials for bone regenerative engineering. Adv Healthc Mater. (2015) 4:1268–85. doi: 10.1002/adhm.201400760 PMC4507442 25846250

[3] Li J Zhou ZX Wen J Jiang F Xia Y. Human amniotic mesenchymal stem cells promote endogenous bone regeneration. Front Endocrinol. (2020) 11:543623. doi: 10.3389/fendo.2020.543623 PMC7562979 33133012

[4] Lian MF Qiao ZG Qiao SC Zhang X Lin JM Xu RD. Nerve growth factor-preconditioned mesenchymal stem cell-derived exosome-functionalized 3D-printed hierarchical porous scaffolds with neuro-promotive properties for enhancing innervated bone regeneration. ACS Nano. (2024) 18:7504–20. doi: 10.1021/acsnano.3c11890 38412232

[5] Mi BB Xiong Y Zha KK Cao FQ Zhou W Abbaszadeh S. Immune homeostasis modulation by hydrogel-guided delivery systems: a tool for accelerated bone regeneration. Biomater Sci. (2023) 11:6035–59. doi: 10.1039/d3bm00544e 37522328

[6] Wu F Li H Jin L Li X Ma Y You J. Deer antler base as a traditional Chinese medicine: A review of its traditional uses, chemistry and pharmacology. J Ethnopharmacol. (2013) 145:403–15. doi: 10.1016/j.jep.2012.12.008 23246455

[7] Baciut M Baciut G Simon V Albon C Coman V Prodan P. Investigation of deer antler as a potential bone regenerating biomaterial. J Optoelectron Adv Mater. (2007) 9:2547–50.

[8] Liu LY Jiao Y Yang M Wu L Long GH Hu W. Network pharmacology, molecular docking and molecular dynamics to explore the potential immunomodulatory mechanisms of deer antler. Int J Mol Sci. (2023) 24:24. doi: 10.3390/ijms241210370 PMC10299714 37373516

[9] Zha EH Li XX Li DD Guo XS Gao SY Yue XQ. Immunomodulatory effects of a 3.2 kDa polypeptide from velvet antler of *Cervus nippon* Temminck. Int Immunopharmacol. (2013) 16:210–3. doi: 10.1016/j.intimp.2013.02.027 23562758

[10] Zhang K Niu L-C Yuan F-J Liu S-P. Research on promotory effect of traditional Chinese medicine on fracture healing in cell and molecular level. Zhongguo gu shang = China J Orthoped Traumatol. (2017) 30:777–82. doi: 10.3969/j.issn.1003-0034.2017.08.021 29455515

[11] Zhao L Zhang H Li N Chen JM Xu H Wang YJ. Network pharmacology, a promising approach to reveal the pharmacology mechanism of Chinese medicine formula. J Ethnopharmacol. (2023) 309:23. doi: 10.1016/j.jep.2023.116306 36858276

[12] Sun W Bai MH Wang J Wang B Liu YX Wang Q. Machine learning-assisted rapid determination for traditional Chinese Medicine Constitution. Chin Med. (2024) 19:14. doi: 10.1186/s13020-024-00992-0 39278905 PMC11403957

[13] Chung MC Su LJ Chen CL Wu LC. y AI-assisted literature exploration of innovative Chinese medicine formulas. Front Pharmacol. (2024) 15:1347882. doi: 10.3389/fphar.2024.1347882 38584602 PMC10995307

[14] Liu ZY Guo FF Wang Y Li C Zhang XL Li HL. BATMAN-TCM: a bioinformatics analysis tool for molecular mechANism of traditional chinese medicine. Sci Rep. (2016) 6:11. doi: 10.1038/srep21146 26879404 PMC4754750

[15] Barrett T Wilhite SE Ledoux P Evangelista C Kim IF Tomashevsky M. NCBI GEO: archive for functional genomics data sets-update. Nucleic Acids Res. (2013) 41:D991–5. doi: 10.1093/nar/gks1193 PMC3531084 23193258

[16] Ritchie ME Phipson B Wu D Hu YF Law CW Shi W. *limma* powers differential expression analyses for RNA-sequencing and microarray studies. Nucleic Acids Res. (2015) 43:13. doi: 10.1093/nar/gkv007 25605792 PMC4402510

[17] Robinson MD McCarthy DJ Smyth GK. edgeR: a Bioconductor package for differential expression analysis of digital gene expression data. Bioinformatics. (2010) 26:139–40. doi: 10.1093/bioinformatics/btp616 PMC2796818 19910308

[18] Johnson WE Li C Rabinovic A. Adjusting batch effects in microarray expression data using empirical Bayes methods. Biostatistics. (2007) 8:118–27. doi: 10.1093/biostatistics/kxj037 16632515

[19] Leek JT Johnson WE Parker HS Fertig EJ Jaffe AE Zhang YS. sva: Surrogate Variable Analysis. Bioconductor. (2025). doi: 10.18129/B9.bioc.sva

[20] Gu ZG Eils R Schlesner M. Complex heatmaps reveal patterns and correlations in multidimensional genomic data. Bioinformatics. (2016) 32:2847–9. doi: 10.1093/bioinformatics/btw313 27207943

[21] The Gene Ontology Consortium. Gene Ontology Consortium: going forward. Nucleic Acids Res. (2015) 43:D1049–56. doi: 10.1093/nar/gku1179 PMC4383973 25428369

[22] Kanehisa M Furumichi M Sato Y Ishiguro-Watanabe M Tanabe M. KEGG: integrating viruses and cellular organisms. Nucleic Acids Res. (2021) 49:D545–d551. doi: 10.1093/nar/gkaa970 33125081 PMC7779016

[23] Yu GC Wang LG Han YY He QY. clusterProfiler: an R package for comparing biological themes among gene clusters. Omics-a J Integr Biol. (2012) 16:284–7. doi: 10.1089/omi.2011.0118 PMC3339379 22455463

[24] Chen H Boutros PC. VennDiagram: a package for the generation of highly-customizable Venn and Euler diagrams in R. BMC Bioinf. (2011) 12:7. doi: 10.1186/1471-2105-12-35 PMC3041657 21269502

[25] Friedman J Hastie T Tibshirani R. Regularization paths for generalized linear models via coordinate descent. J Stat Softw. (2010) 33:1–22. doi: 10.18637/jss.v033.i01 20808728 PMC2929880

[26] Meyer D Dimitriadou E Hornik K Weingessel A Leisch F. Misc functions of the department of statistics, probabilityTheory group (Formerly: E1071). TU Wien (2015). doi: 10.32614/CRAN.package.e1071

[27] Szklarczyk D Gable AL Lyon D Junge A Wyder S Huerta-Cepas J. STRING v11: protein-protein association networks with increased coverage, supporting functional discovery in genome-wide experimental datasets. Nucleic Acids Res. (2019) 47:D607–13. doi: 10.1093/nar/gky1131 PMC6323986 30476243

[28] Warde-Farley D Donaldson SL Comes O Zuberi K Badrawi R Chao P. The GeneMANIA prediction server: biological network integration for gene prioritization and predicting gene function. Nucleic Acids Res. (2010) 38:W214–20. doi: 10.1093/nar/gkq537 PMC2896186 20576703

[29] Robin X Turck N Hainard A Tiberti N Lisacek F Sanchez JC. pROC: an open-source package for R and S plus to analyze and compare ROC curves. BMC Bioinf. (2011) 12:8. doi: 10.1186/1471-2105-12-77 PMC3068975 21414208

[30] Newman AM Liu CL Green MR Gentles AJ Feng WG Xu Y. Robust enumeration of cell subsets from tissue expression profiles. Nat Methods. (2015) 12:453. doi: 10.1038/nmeth.3337 25822800 PMC4739640

[31] Villanueva RAM Chen ZJ. ggplot2: elegant graphics for data analysis, 2nd edition. Measurement-Interdiscip Res Perspect. (2019) 17:160–7. doi: 10.1080/15366367.2019.1565254

[32] Kim S Chen J Cheng TJ Gindulyte A He J He SQ. PubChem in 2021: new data content and improved web interfaces. Nucleic Acids Res. (2021) 49:D1388–95. doi: 10.1093/nar/gkaa971 PMC7778930 33151290

[33] Berman HM Battistuz T Bhat TN Bluhm WF Bourne PE Burkhardt K. The protein data bank. Acta Crystallograph Sect D-Structural Biol. (2002) 58:899–907. doi: 10.1107/s0907444902003451 12037327

[34] Trott O Olson AJ. Software news and update autoDock vina: improving the speed and accuracy of docking with a new scoring function, efficient optimization, and multithreading. J Comput Chem. (2010) 31:455–61. doi: 10.1002/jcc.21334 PMC3041641 19499576

[35] Grunsky EC. R: a data analysis and statistical programming environment - an emerging tool for the geosciences. Comput Geosci. (2002) 28:1219–22. doi: 10.1016/s0098-3004(02)00034-1

[36] Siddiqui JA Partridge NC. Physiological bone remodeling: systemic regulation and growth factor involvement. Physiol (Bethesda). (2016) 31:233–45. doi: 10.1152/physiol.00061.2014 PMC6734079 27053737

[37] Einhorn TA Gerstenfeld LC. Fracture healing: mechanisms and interventions. Nat Rev Rheumatol. (2015) 11:45–54. doi: 10.1038/nrrheum.2014.164 25266456 PMC4464690

[38] Eferl R Wagner EF. AP-1: a double-edged sword in tumorigenesis. Nat Rev Cancer. (2003) 3:859–68. doi: 10.1038/nrc1209 14668816

[39] Zenz R Eferl R Scheinecker C Redlich K Smolen J Schonthaler HB. Activator protein 1 (Fos/Jun) functions in inflammatory bone and skin disease. Arthritis Res Ther. (2008) 10:10. doi: 10.1186/ar2338 PMC2374460 18226189

[40] Stein GS Lian JB Stein JL Van Wijnen AJ Montecino M. Transcriptional control of osteoblast growth and differentiation. Physiol Rev. (1996) 76:593–629. doi: 10.1152/physrev.1996.76.2.593 8618964

[41] Chen ZT Klein T Murray RZ Crawford R Chang J Wu CT. Osteoimmunomodulation for the development of advanced bone biomaterials. Mater Today. (2016) 19:304–21. doi: 10.1016/j.mattod.2015.11.004

[42] Walsh MC Kim N Kadono Y Rho J Lee SY Lorenzo J. Osteoimmunology: interplay between the immune system and bone metabolism. Annu Rev Immunol. (2006) 24:33–63. doi: 10.1146/annurev.immunol.24.021605.090646 16551243

[43] Kovach TK Dighe AS Lobo PI Cui Q. Interactions between MSCs and immune cells: implications for bone healing. J Immunol Res. (2015) 2015:752510. doi: 10.1155/2015/752510 26000315 PMC4427002

[44] Kurotaki D Sasaki H Tamura T. Transcriptional control of monocyte and macrophage development. Int Immunol. (2017) 29:97–107. doi: 10.1093/intimm/dxx016 28379391

[45] Behre G Whitmarsh AJ Coghlan MP Hoang T Carpenter CL Zhang DE. c-Jun is a JNK-independent coactivator of the PU.1 transcription factor. J Biol Chem. (1999) 274:4939–46. doi: 10.1074/jbc.274.8.4939 9988737

[46] Mahon OR Browe DC Gonzalez-Fernandez T Pitacco P Whelan IT Von Euw S. Nano-particle mediated M2 macrophage polarization enhances bone formation and MSC osteogenesis in an IL-10 dependent manner. Biomaterials. (2020) 239:119833. doi: 10.1016/j.biomaterials.2020.119833 32062479

[47] Riera-Sans L Behrens A. Regulation of alphabeta/gammadelta T cell development by the activator protein 1 transcription factor c-Jun. J Immunol. (2007) 178:5690–700. doi: 10.4049/jimmunol.178.9.5690 17442952

[48] Yee MMF Chin KY Ima-Nirwana S Wong SK. Vitamin A and bone health: A review on current evidence. Molecules. (2021) 26:(6). doi: 10.3390/molecules26061757 PMC8003866 33801011

[49] Prior JC. Progesterone as a bone-trophic hormone. Endocr Rev. (1990) 11:386–98. doi: 10.1210/edrv-11-2-386 2194787

[50] Huang C Ogawa R. Mechanotransduction in bone repair and regeneration. FASEB J. (2010) 24:3625–32. doi: 10.1096/fj.10-157370 20505115

[51] Fan J Jahed V Klavins K. Metabolomics in bone research. Metabolites. (2021) 11:(7). doi: 10.3390/metabo11070434 PMC8303949 34357328

[52] Miron RJ Bohner M Zhang Y Bosshardt DD. Osteoinduction and osteoimmunology: Emerging concepts. Periodontol 2000. (2024) 94:9–26. doi: 10.1111/prd.12519 37658591

[53] Lin GL Hankenson KD. Integration of BMP, Wnt, and notch signaling pathways in osteoblast differentiation. J Cell Biochem. (2011) 112:3491–501. doi: 10.1002/jcb.23287 PMC3202082 21793042

