# Correction: Molecular mechanisms of deer antler in promoting osteogenic differentiation of human mesenchymal stem cells via JUN modulation

**DOI:** 10.3389/fimmu.2025.1684846

**Published:** 2025-09-10

**Authors:** Chengcheng Yu, Yinan Wu, Hanxu Huang, Xiumao Li, Jingkai Wang, Chao Liu, Yuanqing Shen, Donghua Huang, Ruofu Tang, Zhan Wang, Lifeng Jiang, Fangcai Li

**Affiliations:** ^1^ Department of Orthopedics, Second Affiliated Hospital, Zhejiang University School of Medicine, Hangzhou, China; ^2^ Zhejiang Cancer Hospital, Institute of Medical Research, Chinese Academy of Sciences, Hangzhou, China; ^3^ Rehabilitation Department, Second Affiliated Hospital, Zhejiang University School of Medicine, Hangzhou, China

**Keywords:** deer antler, osteogenic differentiation, JUN, bioinformatics analysis, machine learning, immune microenvironment modulation

Authors “Chengcheng Yu” and “Yinan Wu” were erroneously omitted as equal contributing authors.

The original version of this article has been updated.

