# Supplementary material for: Molecular mechanisms of deer antler in promoting osteogenic differentiation of human mesenchymal stem cells via JUN modulation
Source: Front Immunol. 2025 May 29;16:1550249. doi: 10.3389/fimmu.2025.1550249 (PMC12158746; doi:10.3389/fimmu.2025.1550249)
Supplement: Supplementary file 2 [file Table1.docx]

**Supplementary Table S1. Overview of GEO Datasets Used for Osteogenic Differentiation Analysis**

| Dataset ID | Sample Size | Cell Source | Culture Conditions | Osteogenic Induction | Platform |
| --- | --- | --- | --- | --- | --- |
| GSE80614 | 15 (3 vs 12) | hMSC (bone marrow) | α-MEM supplemented with 20 mM HEPES, streptomycin/penicillin, 1.8 mM CaCl₂, and 10% heat-inactivated fetal calf serum (HI-FCS), pH 7.5. | 10 mM beta-glycerophosphate, 100 nM dexamethasone | GPL6947 (Illumina) |
| GSE100752 | 52 (19vs 33) | hESC-derived MSCs, fetal limb MSCs | MSCGM BulletKit (Lonza, MD, USA), 37°C, 5% CO₂ | Lonza hMSC Osteogenic Differentiation BulletKits, supplemented with recombinant human TGF-B3. | GPL18405 (Illumina) |
| GSE12267 | 16 (4 vs 12) | hMSC (bone marrow) | α-MEM + 10% FBS + pen-strep | 100 μM Ascorbic Acid-2-Phosphate + 10⁻⁸ M Dexamethasone + 10 mM β-Glycerophosphate. | GPL570 (Affymetrix) |
| GSE28205 | 13 (4 vs 9) | hMSC (bone marrow) | Basal media + 10% FBS + antibiotics | Osteogenic medium induction | GPL6883 (Illumina) |
| GSE9451 | 6 (3 vs 3) | Human iliac mesenchymal stem cells | Low glucose DMEM + 10% FBS | Osteogenic differentiation medium (standard) | GPL570 (Affymetrix) |
| GSE18043 | 12 (3 vs 9) | hMSC (bone marrow) | α-MEM with 10% FCS, 1 ng/mL FGF2, L-glutamine, penicillin | Dexamethasone | GPL570 (Affymetrix) |
| GSE28074 | 18 (8 vs 10) | hMSC from healthy donors | α-MEM + 10% FBS | BMP6 for osteogenesis | GPL13303 (Affymetrix) |
